# Roles of retinoic acid and Tbx1/10 in pharyngeal segmentation: amphioxus and the ancestral chordate condition

**DOI:** 10.1186/2041-9139-5-36

**Published:** 2014-10-09

**Authors:** Demian Koop, Jie Chen, Maria Theodosiou, João E Carvalho, Susana Alvarez, Angel R de Lera, Linda Z Holland, Michael Schubert

**Affiliations:** Marine Biology Research Division, Scripps Institution of Oceanography, University of California San Diego, La Jolla, CA 92093-0202 USA; Institut de Génomique Fonctionnelle de Lyon (CNRS UMR 5242, UCBL, ENS, INRA 1288), Ecole Normale Supérieure de Lyon, 69364 Lyon, Cedex 07, France; Sorbonne Universités, UPMC Université Paris 06, CNRS, UMR 7009, Laboratoire de Biologie du Développement de Villefranche-sur-Mer, Observatoire Océanologique de Villefranche-sur-Mer, 06230 Villefranche-sur-Mer, France; CNRS, UMR 7009, Laboratoire de Biologie du Développement de Villefranche-sur-Mer, Observatoire Océanologique de Villefranche-sur-Mer, 06230 Villefranche-sur-Mer, France; Departamento de Química Orgánica, Universidade de Vigo, 33610 Vigo, Spain

**Keywords:** Cephalochordate, Cyp26 function, evolution of developmental mechanisms, evolution of the vertebrate head, functional knockdown, pharmacological treatments, pharyngeal patterning, retinoic acid signaling, Tbx1/10

## Abstract

**Background:**

Although chordates descend from a segmented ancestor, the evolution of head segmentation has been very controversial for over 150 years. Chordates generally possess a segmented pharynx, but even though anatomical evidence and gene expression analyses suggest homologies between the pharyngeal apparatus of invertebrate chordates, such as the cephalochordate amphioxus, and vertebrates, these homologies remain contested. We, therefore, decided to study the evolution of the chordate head by examining the molecular mechanisms underlying pharyngeal morphogenesis in amphioxus, an animal lacking definitive neural crest.

**Results:**

Focusing on the role of retinoic acid (RA) in post-gastrulation pharyngeal morphogenesis, we found that during gastrulation, RA signaling in the endoderm is required for defining pharyngeal and non-pharyngeal domains and that this process involves active degradation of RA anteriorly in the embryo. Subsequent extension of the pharyngeal territory depends on the creation of a low RA environment and is coupled to body elongation. RA further functions in pharyngeal segmentation in a regulatory network involving the mutual inhibition of RA- and Tbx1/10-dependent signaling.

**Conclusions:**

These results indicate that the involvement of RA signaling and its interactions with Tbx1/10 in head segmentation preceded the evolution of neural crest and were thus likely present in the ancestral chordate. Furthermore, developmental comparisons between different deuterostome models suggest that the genetic mechanisms for pharyngeal segmentation are evolutionary ancient and very likely predate the origin of chordates.

**Electronic supplementary material:**

The online version of this article (doi:10.1186/2041-9139-5-36) contains supplementary material, which is available to authorized users.

## Background

The evolution of head segmentation in chordates has been controversial for well over 150 years. In all chordates (vertebrates, tunicates and amphioxus), the pharynx is segmented into gill slits (aquatic chordates) or pouches (terrestrial chordates) and pharyngeal arches. The pharyngeal arches have a mesodermal core that derives from head mesoderm (or in amphioxus, the anteriormost somites) plus, in vertebrates only, a neural crest component that gives rise to pharyngeal cartilages. The mesoderm of the most anterior pharyngeal arch, the mandibular arch, gives rise to the velar muscle in the lamprey and to the jaw and other head muscles in gnathostomes. In addition to anatomical and fossil evidence [1–4], domains of gene expression have suggested homologies between the amphioxus and vertebrate pharynx as well as between the anterior somites of amphioxus and the head mesoderm of vertebrates. For example, *engrailed* is expressed in the posterior portion of each of the anterior somites of amphioxus and in the posterior wall of the mandibular head cavity and upper lip in the lamprey [5, 6] as well as in the mandibular mesoderm of sharks [7] and the jaw muscles of the zebrafish [8]. Similarly, in amphioxus, *Tbx1/10* is expressed in the somites and in their ventral mesodermal extensions as well as in adjacent endoderm. In the lamprey, the gene is expressed in the mesenchyme of the upper lip, velar muscles and pharyngeal arches, while, in gnathostomes, *Tbx1/10* is detectable both in neural crest and head mesenchyme derivatives [9, 10]. In addition, in both amphioxus and aquatic vertebrates, *Pax2/5/8* is expressed where the gill slits are forming as well as in the endostyle or its vertebrate homolog, the thyroid gland, and in the central nervous system (CNS), while *Pax1/9* genes are broadly expressed in the pharyngeal endoderm of all chordates [11–13].

We have previously defined an early phase of pharyngeal specification in amphioxus, which occurs during the gastrula stage and is regulated by retinoic acid (RA) signaling [14–17]. RA, a natural morphogen synthesized from vitamin A, binds to heterodimers of the retinoic acid receptor (RAR) and retinoid X receptor (RXR), allowing the complex to bind to regulatory regions of target genes and thereby activate transcription. The gene encoding the RA-degrading enzyme Cyp26-2 is expressed anterior to the anteriormost domain of *Tbx1/10*, indicating considerable reduction of RA signaling in the rostral head [18]. High RA signaling levels in the middle third of the endoderm of early embryos of amphioxus and vertebrates specify the midgut, while low levels of RA signaling in the pharynx specify the pharyngeal endoderm [14–16, 19–22]. Thus, in amphioxus, exogenous RA applied during the gastrula stage causes loss of the pharynx and of all pharyngeal structures by respecifying the pharyngeal endoderm as midgut [14–17]. Similarly, in vertebrates, RA signaling is required for the formation of pharyngeal pouches caudal to the second pharyngeal arch. Thus, excess RA leads to a compression of the pharynx in lampreys [23, 24] and to a fusion of the first two pharyngeal arches in gnathostomes, while the inhibition of RA signaling in gnathostomes, either genetically or by vitamin A deficiency, results in a loss of posterior pharyngeal structures [19–22, 25–27].

In amphioxus, very few direct targets of RA signaling at the gastrula have been identified to date. Of more than 40 genes tested, the only direct targets were *Hox* genes (*Hox1*, *Hox3*), normally expressed in the dorsal/posterior mesendoderm and ectoderm, and *FoxA2-1*, normally expressed in the dorsal/posterior and anterior/ventral mesendoderm. The domains of all three genes are expanded anteriorly in RA-treated embryos [17]. Furthermore, knockdown of *Hox1* showed that it mediates the effect of RA in establishing the posterior limit of the pharynx [16]. Indirect targets include *Otx*, which is normally expressed in the anterior mesendoderm and dorsal/anterior ectoderm at the gastrula stage, and *Pax1/9*, which turns on in the pharyngeal endoderm at the very early neurula stage [11, 17].

**Figure 1:**
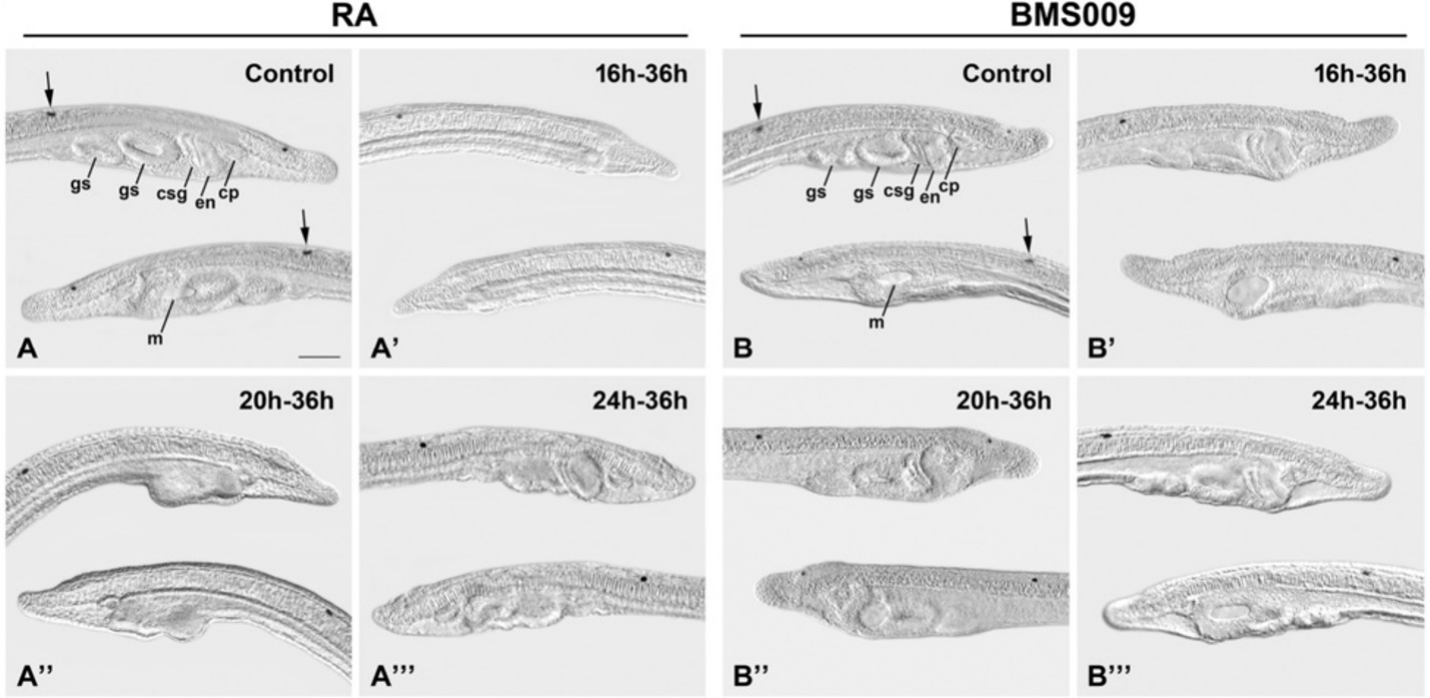
**Exposure to retinoic acid (RA) or the RA receptor (RAR) antagonist BMS009 disrupts patterning and formation of the amphioxus pharynx.** The anterior portions of amphioxus larvae at 36 hours post fertilization (hpf) are shown in lateral views. The right side of the larva is shown on top (with anterior to the right), with the two gill slits (gs), club-shaped gland (csg), endostyle (en) and ciliated pit (cp) in focus, and the left side is shown below (with anterior to the left), with the open mouth (m) in focus. Arrows highlight the first photoreceptor and associated pigment cell in the central nervous system. The 50-μm scale bar in A is applicable to the entire figure. Control larvae at 36 hpf are shown **(A)**, **(B)**, as are 36-hpf larvae treated with 10^-6^ M RA from 16 hpf **(A’)**, 20 hpf **(A”)** and 24 hpf **(A”’)** or 2 × 10^-6^ M BMS009 from 16 hpf **(B’)**, 20 hpf **(B”)** and 24 hpf **(B”’)**.

In normal embryos and larvae of amphioxus, pharyngeal structures are asymmetrically arrayed (Figure 1). By the early larval stage, from anterior to posterior, there is on the right, the endostyle, homologous to the thyroid, the club-shaped gland, a larval secretory structure that undergoes apoptosis at metamorphosis [28], and two gill slits. Additional gill slits form sequentially from anterior to posterior. On the left, the ciliated pit, which will form part of the homolog of the adenohypophysis, forms just anterior to the mouth, thought to be a modified gill slit [29]. The posterior limit of the pharynx in the early larva is at the same anterior/posterior position as the first photoreceptor and associated pigment cell that form in the nerve cord at the level of somite 5. The position of this pigment spot is unaffected by altered RA signaling even though excess RA shifts the *Hox1* domains in the CNS and endoderm anteriorly [30].

Morphological effects of excess RA added at the gastrula stage are first apparent in the early neurula. In untreated embryos, the pharyngeal endoderm marker *Pax1/9* is downregulated where the first gill slit will form [11]. As neurulation proceeds, *Pax1/9* is also downregulated in the primordium of the second gill slit. In embryos treated with RA from the gastrula stage, *Pax1/9* is not downregulated in the gill slit primordia, and the posterior limit of its domain is shifted anteriorly. Similarly, the posterior limit of the normally broad domain of *Otx* in the endoderm shifts anteriorly in RA-treated larvae. Thus, in embryos treated with 10^-6^ M RA at the gastrula stage, much of the pharyngeal endoderm is respecified as midgut [14, 16]. Knockdown experiments showed that Hox1 acts upstream of *Otx* and *Pax1/9* in setting the posterior limit of the pharynx [16]. However, gill slits do form in most embryos when RA addition is delayed until the early neurula stage [14]. Even so, in untreated neurulae, the competitive RA signaling inhibitor *TR2/4* turns on in the gill slit primordia just before the gill slits begin to penetrate [15], suggesting that RA signaling may have to be continuously repressed during the neurula stage to ensure proper development of the gill slits.

To determine if in amphioxus (*Branchiostoma floridae*), an animal lacking definitive neural crest, RA affects pharyngeal morphogenesis post-gastrulation, we adopted a multi-pronged approach. We first manipulated RA signaling in amphioxus embryos from neurula through early larval stages and, as RA and Tbx1/10 mutually repress one another in vertebrates [10], compared the effects to those resulting from loss of Tbx1/10 function. In addition, we determined whether RA suffices to inhibit specification of pharyngeal endoderm or the formation of pharyngeal structures in amphioxus embryos and larvae with reduced Hox1 function. Finally, to comprehensively assess the roles of RA degradation in the developing anterior endoderm, we inhibited Cyp26 enzyme function starting at the gastrula stage. Taken together, our results show that RA signaling has both early and late effects on pharyngeal patterning in amphioxus. During the neurula stage, specification of the pharyngeal endoderm gradually becomes refractory to RA signaling. However, while Cyp26 functions in the anteriormost pharynx to keep RA levels low, partitioning of the pharynx into gill slits is regulated during the neurula stage by fine-tuning of RA levels. Knockdown of Tbx1/10 in the pharyngeal arches has a similar effect as adding RA during the neurula stage, indicating that, in the absence of neural crest, mutual inhibition of RA signaling and Tbx1/10 function is required for the partitioning of the amphioxus pharynx. These results show that the genetic mechanism involving RA and Tbx1/10 for partitioning the pharynx into pharyngeal pouches preceded the evolution of neural crest and was likely present in the ancestral chordate.

## Methods

### Embryo rearing, RA, RAR antagonist (BMS009) and Cyp26 inhibitor (R115866) treatments

Ripe males and females of the Florida amphioxus (*Branchiostoma floridae*) were collected by shovel and sieve in Tampa Bay, Florida (USA), during the summer breeding season. Spawning was induced electrically, and the embryos and larvae were cultured in the laboratory at 29°C as previously described [31]. Stock solutions of all-*trans* RA or the RAR antagonist BMS009, dissolved in dimethyl sulfoxide (DMSO), were added to cultures at different time points, 16 hours post fertilization (hpf), 20 hpf and 24 hpf, at final concentrations of 10^-6^ M and 2 × 10^-6^ M, respectively. Control treatments were 1:1,000 dilutions of DMSO alone [14, 15]. Dishes were incubated in the dark, because RA and BMS009 are light sensitive. Samples were collected at 36 hpf and fixed for *in situ* hybridization as described below. Cyp26 function was inhibited using R115866 (provided by Janssen Research & Development, a division of Janssen Pharmaceutica NV, Beerse, Belgium). A 10^-3^ M stock solution in DMSO was added to embryo cultures at the onset of gastrulation (3.5 hpf) to a final concentration of 5 × 10^-7^ M, and embryos were fixed at 36 hpf.

### Microinjection-based experiments

Microinjection of amphioxus eggs was performed as previously described [31]. Unfertilized eggs were injected with either a control antisense morpholino oligonucleotide (MO) (5’-CCTCTTACCTCAGTTACAATTTATA-3’) or one specific for *AmphiHox1* from *B. floridae* (5’-ATTCTTGCCGTGTCCATTTGCTCCA-3’) or *AmphiTbx1/10* from *B. floridae* (5’-ATAGCGGACTGTTGGCTTCCATGTC-3’) (Gene Tools, Philomath, OR, USA). The activity of both MOs was confirmed by *in vitro* translation assays of the *AmphiHox1*[16] and *AmphiTbx1/10* (Additional file 1: Figure S1) coding regions using the TnT Quick Coupled Transcription/Translation System (Promega, Madison, WI, USA) and a detection system based on the Transcend Non-Radioactive Translation Detection Systems ( Promega, Madison, WI, USA) [16]. Approximately 2 pl of a solution containing 15% glycerol, 2 mg/ml Texas Red dextran (Molecular Probes, Eugene, OR, USA) and 500 μM (*Hox1* and control) or 1,000 μM (*Tbx1/10* and control) MO was injected. Following injection, the eggs were fertilized, cultured and fixed at the early larval stage (36 hpf). For control MO/10^-7^ M RA and *Hox1* MO/10^-7^ M RA treatments, injected embryos were treated with RA at final concentrations of 10^-7^ M continuously from the onset of gastrulation (3.5 hpf). Fixed, injected embryos showing clear fluorescence of the Texas Red dextran were analyzed by *in situ* hybridization [32]. Injection of the control MO at both 500 μM and 1000 μM did not induce any abnormalities.

### Developmental gene expression analyses using *in situ*hybridization

For *in situ* hybridization, samples were fixed according to established protocols [32]. Effects of treatments and MO injections on pharyngeal development were assayed by *in situ* hybridization with antisense riboprobes synthesized for the following genes: *AmphiPax1/9* (U20167) [11], *AmphiSix1/2* (EF195742) [13], *AmphiTbx1/10* (AF262562) [33], *AmphiPax2/5/8* (AF053762) [12], *AmphiPitx* (AJ438768) [34], *AmphiTR2/4* (AF378828) [15] and *AmphiCyp26-2* (EST clone bfne112a21). After *in situ* hybridization, the embryos were photographed as whole mounts using differential interference contrast (DIC) microscopy [32].

## Results

### RA signaling is required for both regional specification and morphogenesis of the pharynx

To determine if RA regulates development of the pharynx once it is initially specified, embryos were treated with either RA or the RAR antagonist BMS009 at the early/mid neurula (16 hpf), mid neurula (20 hpf) and late neurula (24 hpf) stage and subsequently fixed at the early larval stage (36 hpf). Given that the position of the first photoreceptor and associated pigment cell in the nerve cord is unaffected by changing RA signaling levels and that its anterior/posterior position coincides with the posterior limit of the pharynx of the early larva [30], we used this first pigment spot as a landmark to assess changes in pharyngeal length resulting from the treatments.

**Figure 2:**
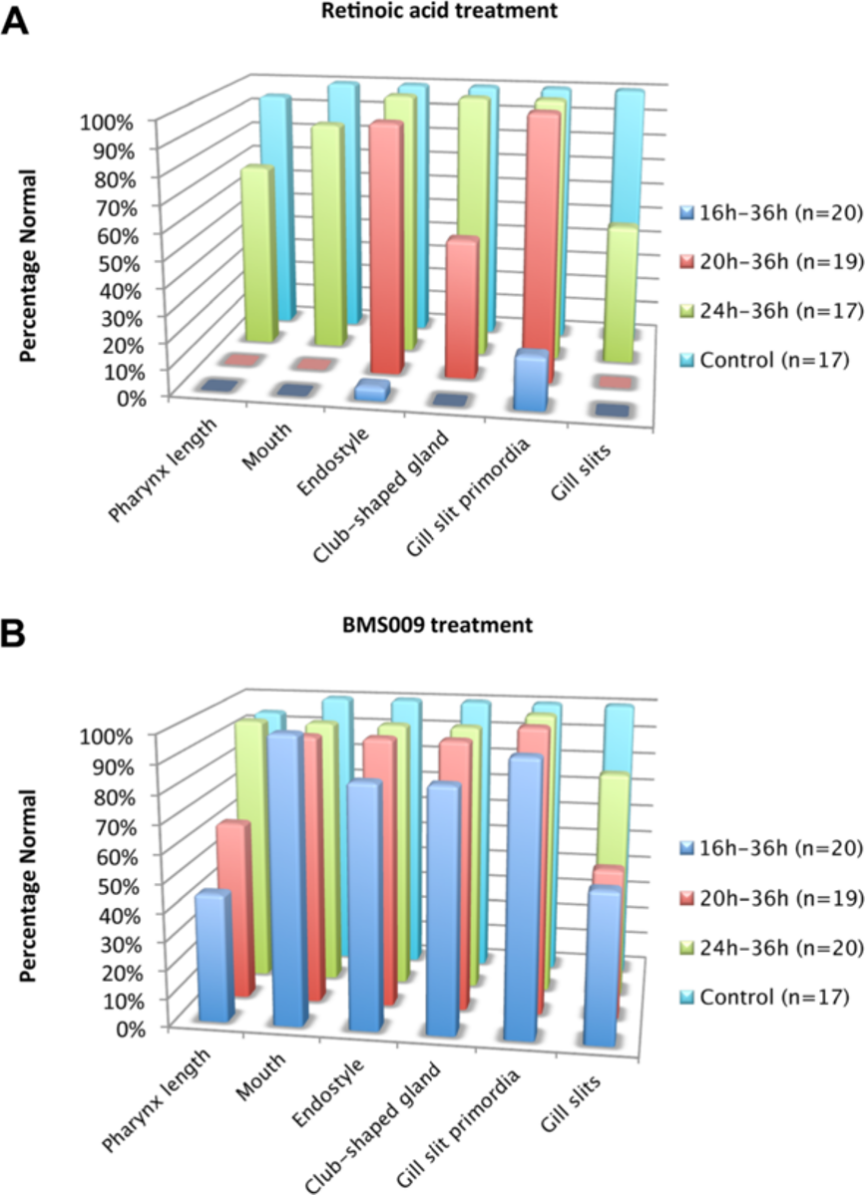
**Stage-dependent effects of retinoic acid (RA) and RA receptor (RAR) antagonist (BMS009) on the formation of pharyngeal structures in amphioxus.** For each feature, the percentage of normal larvae at 36 hours post fertilization (hpf) is indicated. Animals were treated with RA **(A)** or BMS009 **(B)** from 16 hpf, 20 hpf or 24 hpf.

We found that excess RA added at progressively later stages resulted in progressively less severe effects on pharyngeal development (Figure 1). As treated embryos were kept in the dark to avoid degradation of RA, the effects we observed were more severe than in our previous study [14]. RA applied continuously from 16 hpf eliminated all pharyngeal structures (Figure 1A, A’; Figure 2A; Additional file 2: Table S1), indicating that the pharyngeal endoderm had been respecified as midgut (Figure 1A, A’). The ciliated pit was sometimes present (Figure 1A, A’). In embryos treated at 20 hpf, there was an endostyle and sometimes a club-shaped gland as well as a single gill slit primordium lacking a gill slit (Figure 1A, A”; Figure 2A; Additional file 2: Table S1). However, the posterior limit of the pharynx was still far anterior to the first photoreceptor in the CNS (Figure 1A). In embryos treated at 24 hpf, there was a mouth, an endostyle, a club-shaped gland and typically a single gill slit primordium, penetrated in about 50% of the larvae by an abnormal gill slit (Figure 1A, A”’; Figure 2A; Additional file 2: Table S1). A second gill slit primordium was occasionally present, but was less developed compared to controls.

Treatments with the RAR antagonist BMS009 at 16 hpf and 20 hpf resulted in a posterior expansion of the pharynx in 40 to 60% of the larvae. Most larvae had gill slit primordia, but about half of the associated gill slits were smaller than normal (Figure 1B, B’ , B”; Figure 2B; Additional file 2: Table S1). An endostyle and club-shaped gland were typically present, and, as in larvae treated with BMS009 from the gastrula stage, the mouth was often larger than normal (Figure 1B, B’ , B”) [14]. When applied at 24 hpf, BMS009 did not affect the length of the pharynx. The club-shaped gland and endostyle were normal, and the gill slits and mouth were present, although sometimes the mouth was slightly enlarged (Figures 1B, B”’; Figure 2B; Additional file 2: Table S1).

**Figure 3:**
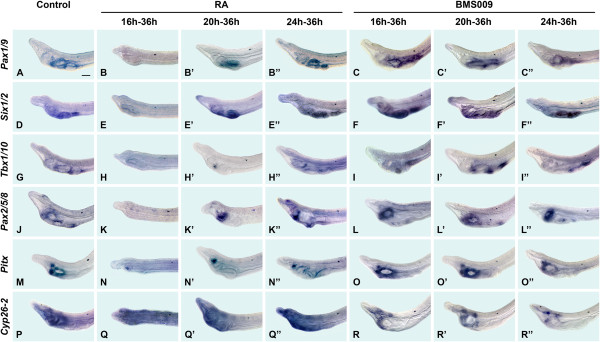
**Molecular characterization of retinoic acid (RA) signaling functions during amphioxus pharyngeal development.** Lateral views of the anterior portions of amphioxus larvae at 36 hours post fertilization (hpf) are shown with the anterior to the left. The 50-μm scale bar in **A** is applicable to the entire figure. Control larvae are shown as well as those treated with either 10^-6^ M RA or 2 × 10^-6^ M BMS009, an RAR antagonist, starting at three different developmental stages (16 hpf, 20 hpf and 24 hpf). The assayed genes are amphioxus *Pax1/9* **(A)**, **(B)**, **(B’)**, **(B”)**, **(C)**, **(C’)**, **(C”)**, *Six1/2* **(D)**, **(E)**, **(E’)**, **(E”)**, **(F)**, **(F’)**, **(F”)**, *Tbx1/10* **(G)**, **(H)**, **(H’)**, **(H”)**, **(I)**, **(I’)**, **(I”)**, *Pax2/5/8* **(J)**, **(K)**, **(K’)**, **(K”)**, **(L)**, **(L’)**, **(L”)**, *Pitx* **(M)**, **(N)**, **(N’)**, **(N”)**, **(O)**, **(O’)**, **(O”)** and *Cyp26-2* **(P)**, **(Q)**, **(Q’)**, **(Q”)**, **(R)**, **(R’)**, **(R”)**. RA treatment at 16 hpf eliminates the expression of *Pax1/9*, *Six1/2*, *Tbx1/10* and *Pax2/5/8* and severely reduces that of *Pitx*. In contrast, expression of *Cyp26-2* is expanded. When RA is applied at progressively later stages, normal expression of the genes is gradually restored. Treatments with BMS009 result in an expansion of the *Pax1/9* domain, in abnormal *Tbx1/10* expression, in a reduction of the *Pax2/5/8* signal in gill slit primordia and in a severe reduction of *Cyp26-2* expression. In contrast, the patterns of *Six1/2* and *Pitx* are largely unaffected. The effects of BMS009 are independent of the treatment time point.

The expression domains of *Pax1/9* and *Tbx1/10* were eliminated by RA treatment at 16 hpf, as were those of *Six1/2* and *Pax2/5/8*, both of which are normally expressed in the gill slit primordia (Figure 3A, B, D, E, G, H, J, K). Expression of *Pitx* around the mouth, in the ciliated pit and in the club-shaped gland was severely reduced (Figure 3M, N). In contrast, expression of *Cyp26-2* was expanded throughout the pharynx, suggesting that this gene is positively regulated by RA (Figure 3P, Q). When RA was applied at progressively later times, normal expression of all these genes was gradually restored. In embryos treated with RA at 20 hpf, *Pax1/9* and *Six1/2* were expressed in association with the single gill slit primordium, while *Tbx1/10* was expressed in a small area just anterior to the gill slit (Figure 3A, B’ , D, E’ , G, H’). Expression of *Pax2/5/8* was limited to the region around the mouth, while expression of *Pitx* in the club-shaped gland was unaffected (Figure 3J, K’ , M, N’). *Cyp26-2* expression was still expanded, but no longer extended posteriorly beyond the first pigment spot (Figure 3P, Q’). In larvae treated with RA from 24 hpf, most expression domains were normal (Figure 3A, B”, D, E”, G, H”, J, K”, M, N”, P, Q”). However, *Tbx1/10* expression between the gill slits was still reduced (Figure 3G, H”), while *Pitx* expression remained restricted to the ciliated pit (Figure 3M, N”), and the domain of *Cyp26-2* was still expanded somewhat posteriorly (Figure 3P, Q”).

Treatment with the RAR antagonist BMS009 had a milder effect than exogenous RA. Although BMS009 expanded expression of *Pax1/9* slightly posteriorly in the pharyngeal endoderm at all times of treatment (Figure 3A, C, C’ , C”), expression of *Six1/2* was largely unaffected (Figure 3D, F, F’ , F”). *Tbx1/10* expression was abnormal at all time points, in agreement with the gill slits being misshapen (Figure 3G, I, I’ , I”). Similarly, *Pax2/5/8* expression was reduced in the gill slit primordia of larvae treated at 16 hpf, 20 hpf and 24 hpf, although the domain around the mouth was unaffected (Figure 3J, L, L’ , L”). Expression of *Pitx* was largely unaffected by BMS009 (Figure 3M, O, O’ , O”). In contrast, *Cyp26-2* expression was severely reduced, although not completely eliminated, by BMS009, which suggests that RA positively regulates *Cyp26-2* (Figure 3P, R, R’ , R”).

Taken together, these results indicate that RA signaling is not only required in early development for regionalization of the endoderm, but is also necessary during the neurula stage for patterning gill slits and mouth. Thus, there appears to be both an early and a late phase for RA signaling: the first requiring low levels for pharyngeal specification and the second requiring localized regulation of RA for patterning within the pharynx.

### Cyp26 activity is required for pharyngeal patterning in amphioxus

Since a low level of RA signaling is required in the amphioxus pharynx for normal specification and patterning, endogenous RA levels must be very tightly regulated. Cyp26 enzymes function as RA sinks by degrading RA into biologically inactive metabolites [35]. Of the three duplicates of amphioxus *Cyp26*[36]*,* one, *Cyp26-2,* is expressed in embryos and early larvae in the anterior CNS, ectoderm, mesoderm and endoderm as well as at the extreme posterior of the animal [18]. At 36 hpf, *Cyp26-2* is broadly expressed anteriorly in the anterior somites, the ectoderm surrounding the mouth, the anterior/ventral endoderm, the endostyle and the club-shaped gland (Figure 3P). The domain of *Cyp26-2* (Figure 3P) in the pharyngeal endoderm is just anterior to that of *Tbx1/10* in the first pharyngeal arch (Figure 3G).

**Figure 4:**
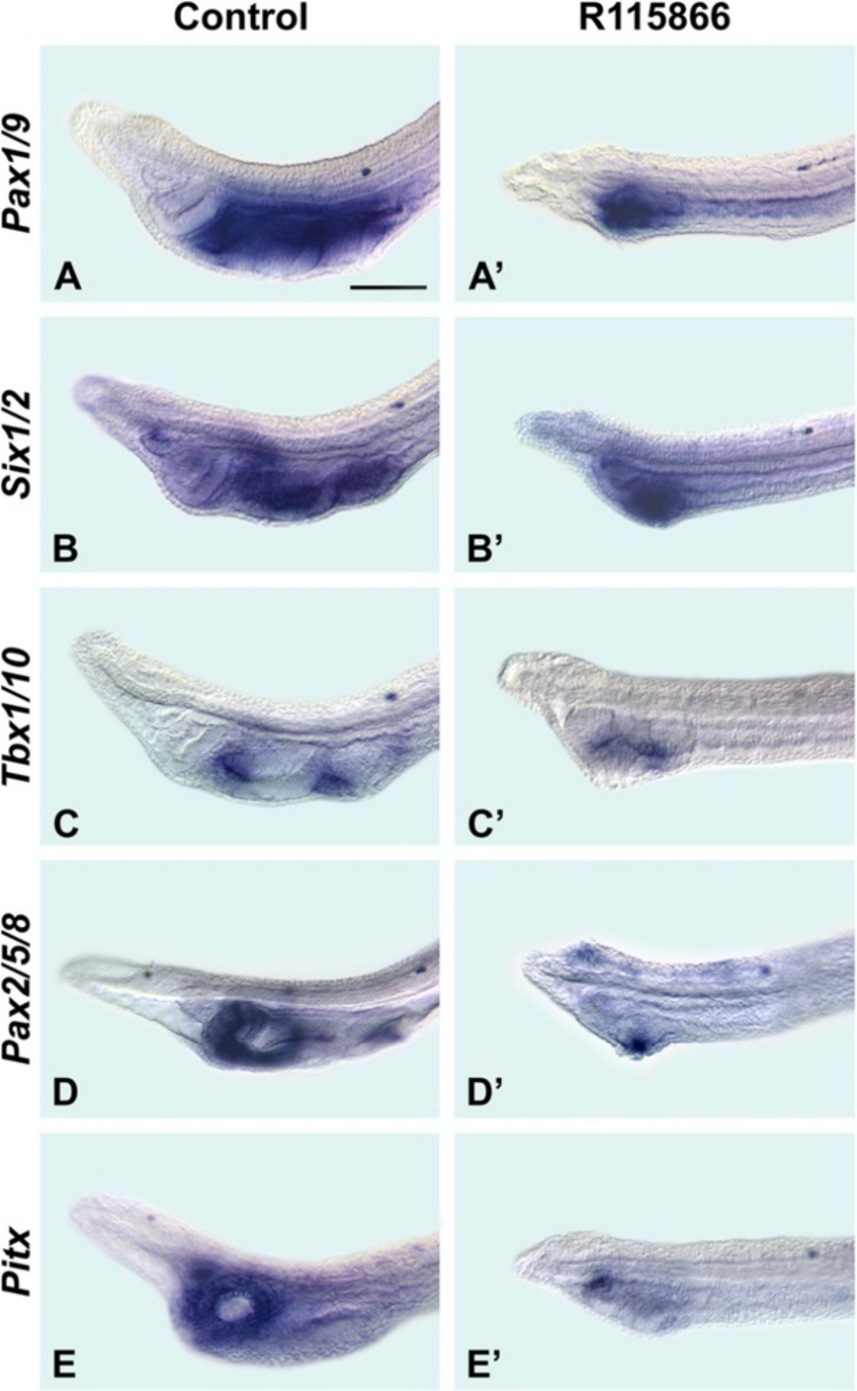
**Cyp26 function is required for patterning the amphioxus pharynx.** The anterior portions of larvae at 36 hours post fertilization (hpf) are shown in lateral views (with anterior to the left). The 50-μm scale bar in **(A)** is applicable to the entire figure. Control larvae are shown as well as those treated with 5 × 10^-7^ M R115866 (a Cyp26 inhibitor) at the onset of gastrulation. The assayed genes are amphioxus *Pax1/9* **(A)**, **(A’)**, *Six1/2* **(B)**, **(B’)**, *Tbx1/10* **(C)**, **(C’)**, *Pax2/5/8* **(D)**, **(D’)** and *Pitx* **(E)**, **(E’)**.

To assess the importance of Cyp26 enzymes in amphioxus pharyngeal development, we treated amphioxus embryos from the onset of gastrulation with the Cyp26 inhibitor R115866 [37]. Similar to treatments with RA, inhibition of Cyp26 caused a dramatic shortening of the pharynx and loss of pharyngeal structures: while 21 of 21 control larvae developed normally, 35 of 35 treated larvae showed pharyngeal abnormalities. Lost structures included the mouth, the endostyle, the club-shaped gland and one gill slit; however, a single gill slit primordium was present (Figure 4). *Pax1/9* expression was compressed anteriorly, being restricted chiefly to the level of the gill slit primordium (Figure 4A, A’), as were the domains of *Six1/2* (Figure 4B, B’) and *Pax2/5/8* (Figure 4D, D’). *Tbx1/10* was weakly expressed anterior and posterior to the single gill slit primordium (Figure 4C, C’), while *Pitx* was only expressed in the ciliated pit (Figure 4E, E’).

Together, these data indicate that inhibition of Cyp26 function leads to the loss of anterior pharyngeal structures, including the mouth, the endostyle, the club-shaped gland and one gill slit. These findings suggest that repression of RA signaling by Cyp26 activity is required to protect the anteriormost part of the amphioxus pharynx from RA teratology.

### RA-dependent regionalization of the endoderm requires Hox1 function, while RA-dependent pharyngeal morphogenesis does not

To separate RA signaling functions in endoderm regionalization from those in pharyngeal morphogenesis, we assessed the effects of RA on pharyngeal development in a Hox1-reduced environment. As noted above, Hox1 mediates the roles of RA in establishing the posterior limit of the pharynx [16, 17]. Knockdown of Hox1 function results in a posterior expansion of the pharynx, which is similar to the effect obtained by treatment with BMS009 at the gastrula stage [16]. Combination of RA treatments with the knockdown of Hox1 function may thus help reveal roles of RA signaling in pharyngeal development, which are independent of its initial, Hox1-mediated role in establishing the posterior limit of the pharynx.

**Figure 5:**
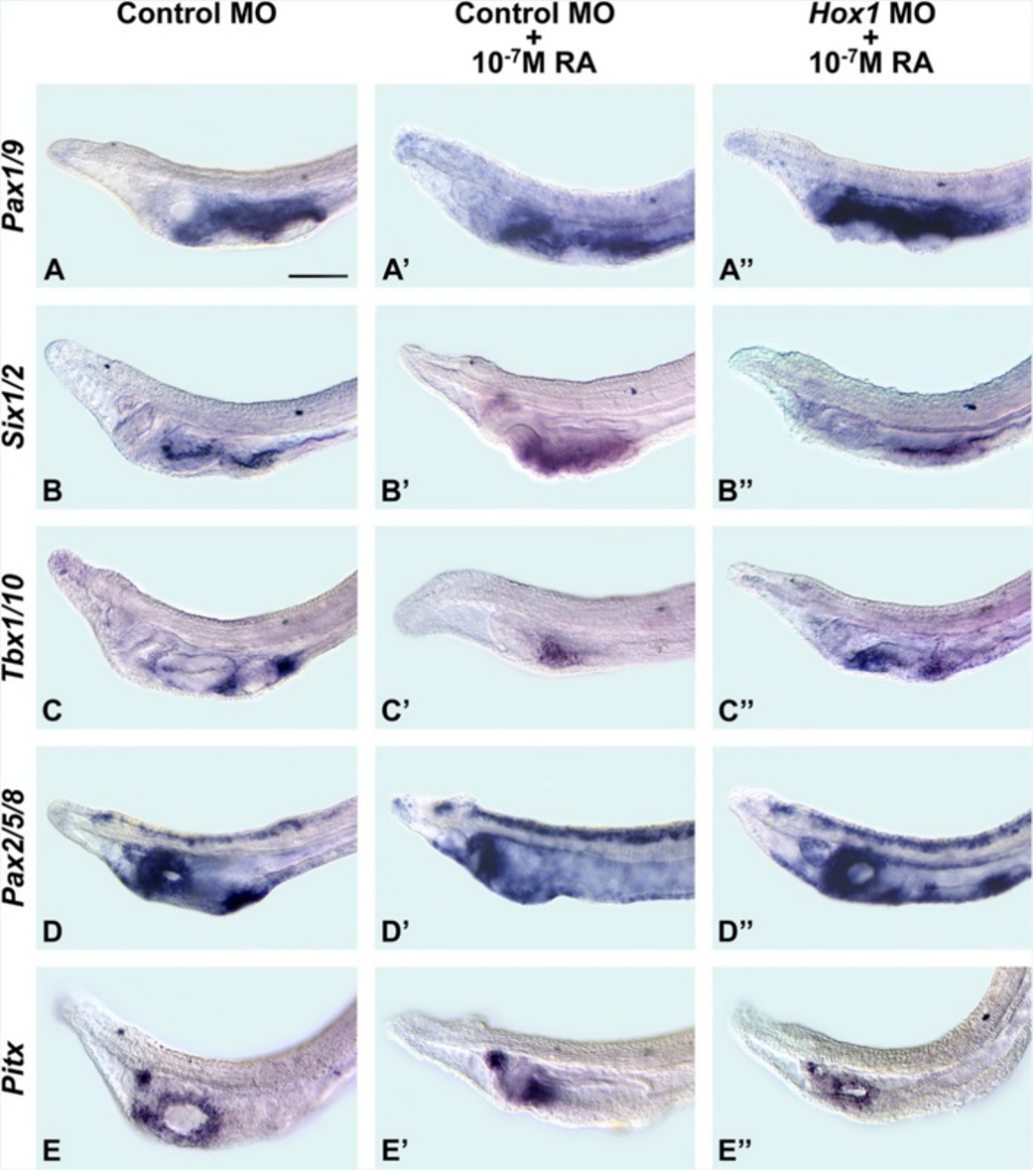
**Amphioxus pharyngeal development depends on retinoic acid (RA) signaling for both anterior-posterior regionalization and morphogenesis.** The anterior portions of larvae at 36 hours post fertilization (hpf) are shown in lateral views (with anterior to the left). The 50-μm scale bar in **(A)** is applicable to the entire figure. Amphioxus larvae are shown that were injected before fertilization with either 500 μM control antisense morpholino oligonucleotide (MO) or with 500 μM control MO followed by a treatment with 10^-7^ M RA (at the onset of gastrulation) or with 500 μM *Hox1* MO followed by treatment with 10^-7^ M RA (at the onset of gastrulation). The assayed genes are amphioxus *Pax1/9* **(A)**, **(A’)**, **(A”)**, *Six1/2* **(B)**, **(B’)**, **(B”)**, *Tbx1/10* **(C)**, **(C’)**, **(C”)**, *Pax2/5/8* **(D)**, **(D’)**, **(D”)** and *Pitx* **(E)**, **(E’)**, **(E”)**.

In larvae injected with the control MO and treated with 10^-7^ M RA, the pharynx was evidently specified, but gill slits did not form and *Pax1/9* expression was downregulated only in a small domain ventrally in the pharynx (Figure 5A, A’). In larvae with combined *Hox1* MO injections and 10^-7^ M RA treatment, there were two ventral zones of reduced *Pax1/9* expression, indicating that two gill slit primordia were specified (Figure 5A, A”). In embryos injected with the control MO and treated with 10^-7^ M RA, there was a single domain of *Six1/2* ventrally in the pharyngeal endoderm, suggesting that the two gill slit primordia were fused (Figure 5B, B’). This domain was expanded posteriorly in embryos injected with *Hox1* MO and treated with 10^-7^ M RA. However, in these larvae, the signal was still continuous and not in two discrete patches, suggesting that the two gill slit primordia remained fused (Figure 5B”). Expression of *Tbx1/10* between the gill slits was also disrupted by RA (Figure 5C, C’), while injection of *Hox1* MO and treatment with 10^-7^ M RA resulted in ectopic expression of *Tbx1/10* ventrally in the pharynx (Figure 5C, C”).

Treatments with 10^-7^ M RA reduced the *Pax2/5/8* signal around the mouth and in the gill slits, but not in the endostyle or CNS (Figure 5D’). Combined *Hox1* MO injection and RA treatment largely restored the mouth- and gill slit-associated domains*.* In fact, the domain of *Pax2/5/8* in the gill slit region was considerably expanded (Figure 5D”). In RA-treated larvae, expression of *Pitx* was restricted to the ciliated pit plus a small cluster of ectodermal cells, indicating that 10^-7^ M RA is sufficient to suppress mouth formation (Figure 5E, E’), as has previously been described [14, 16]. In contrast, in *Hox1* MO/10^-7^ M RA larvae, *Pitx* expression surrounds a relatively small mouth (Figure 5E”).

Importantly, in larvae treated with 10^-7^ M RA, which induced severe pharyngeal defects in only 31 of 50 control MO-injected larvae, the injection of *Hox1* MO seemed to preferentially rescue the expression of pharyngeal regionalization markers (in 13 of 13 larvae), while expression of genes marking specific pharyngeal structures tended to be only partially restored (in 23 of 34 larvae). Injection of the control MO alone did not induce any developmental abnormalities (in 45 of 50 larvae). Altogether, these experiments are in agreement with the idea that, in amphioxus, RA signaling initially acts via Hox1 to control anterior-posterior regionalization of the endoderm and subsequently assumes roles in pharyngeal morphogenesis that are independent of Hox1.

### Tbx1/10 is involved in segmentation of the pharyngeal endoderm and patterning of the gill slit primordia in amphioxus

**Figure 6:**
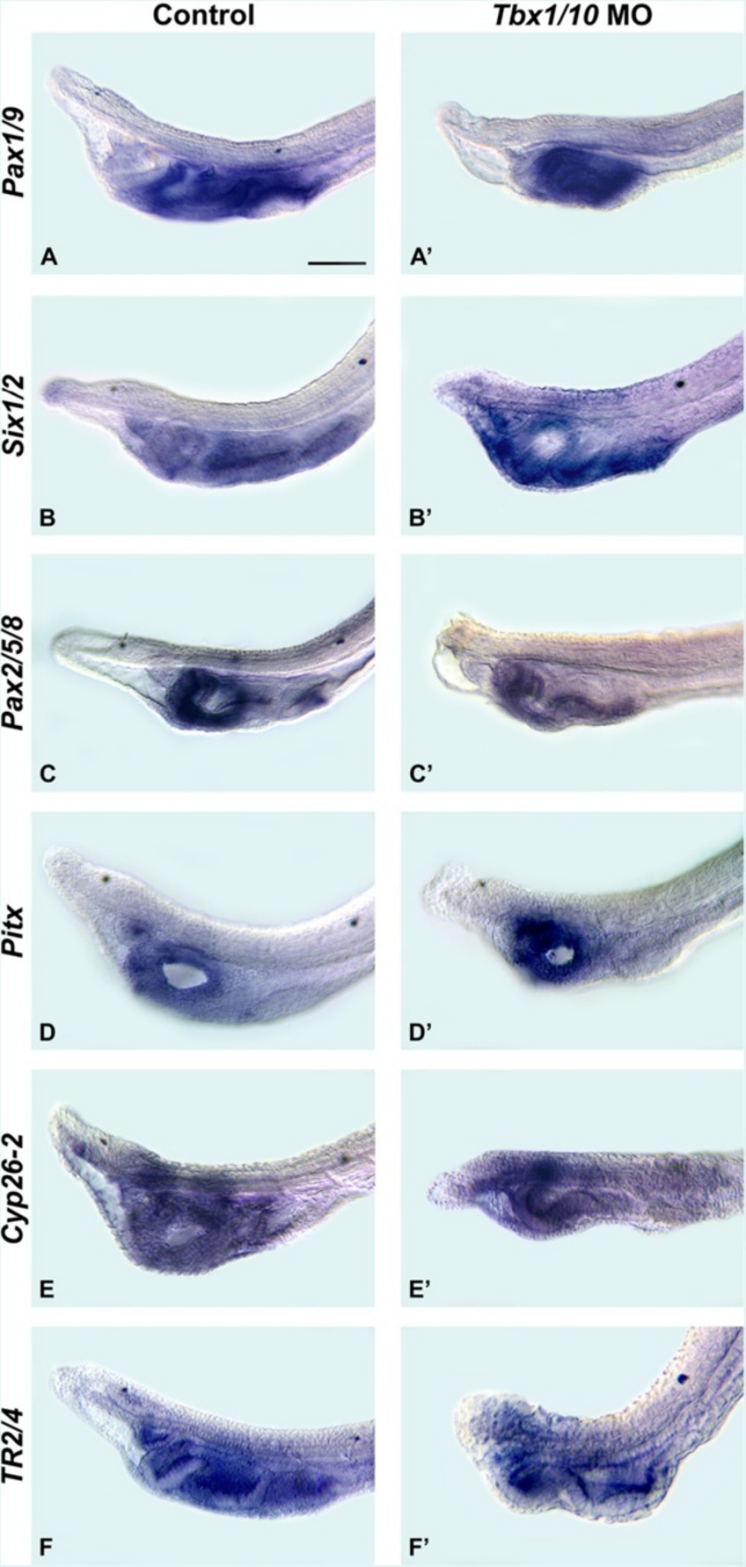
**Tbx1/10 function is required for gill slit formation in the amphioxus pharynx.** The anterior portions of larvae at 36 hours post fertilization (hpf) are shown in lateral views (with anterior to the left). The 50-μm scale bar in **(A)** is applicable to the entire figure. Amphioxus larvae are shown that were injected before fertilization with either 1,000 μM control antisense morpholino oligonucleotide (MO) or with 1,000 μM *Tbx1/10* MO. The assayed genes are amphioxus *Pax1/9* **(A)**, **(A’)**, *Six1/2* **(B)**, **(B’)**, *Pax2/5/8* **(C)**, **(C’)**, *Pitx* **(D)**, **(D’)**, *Cyp26-2* **(E)**, **(E’)** and *TR2/4* **(F)**, **(F’)**.

Since, in amphioxus, *Tbx1/10* is expressed in mesoderm and endoderm between adjacent gill slits [33], we knocked down *Tbx1/10* function to test whether it might be involved in RA-dependent segmentation of the pharynx. Larvae injected with a *Tbx1/10* MO exhibited a smaller pharynx and severely malformed and fused gill slit primordia (for embryos injected with the control MO, 10 of 10 were normal, while 22 of 30 embryos injected with the *Tbx1/10* MO had pharyngeal abnormalities) (Figure 6). The domain of *Pax1/9* expression was reduced, which was largely due to the shorter pharynx (Figure 6A, A’). The *Six1/2* signal was disorganized, in agreement with abnormal gill slit primordia that are elongated and possibly fused (Figure 6B, B’). This elongation and fusion is most apparent in larvae labeled with *Pax2/5/8,* as it marks where the gill slits will form (Figure 6C, C’). Expression of *Pax2/5/8* in the endostyle and mouth is not altered in *Tbx1/10* MO-injected larvae (Figure 6C, C’). *Pitx* expression remains detectable around the open mouth as well as in the club-shaped gland (Figure 6D, D’). In contrast, pharyngeal expression of *Cyp26-2* is disorganized and somewhat reduced in *Tbx1/10* MO-injected larvae (Figure 6E, E’), as is that of *TR2/4* (Figure 6F, F’), which is normally expressed in the pharyngeal endoderm, most conspicuously in mouth and gill slit primordia [15]. Importantly, after *Tbx1/10* knockdown, *TR2/4* expression remains detectable in the mouth as well as in the primordia of the abnormal gill slits (Figure 6F, F’).

These data indicate that Tbx1/10 functions in separating gill slit primordia in the developing amphioxus pharynx. The phenotypes obtained from *Tbx1/10* knockdown are reminiscent of those obtained by RA treatments in a *Hox1* knockdown context, suggesting a mutual inhibition of RA signaling and Tbx1/10 function in amphioxus pharyngeal development, a feature previously proposed for vertebrate pharyngeal arch and cardiovascular development [10].

## Discussion

### RA signaling functions during distinct phases of amphioxus pharyngeal development

**Figure 7:**
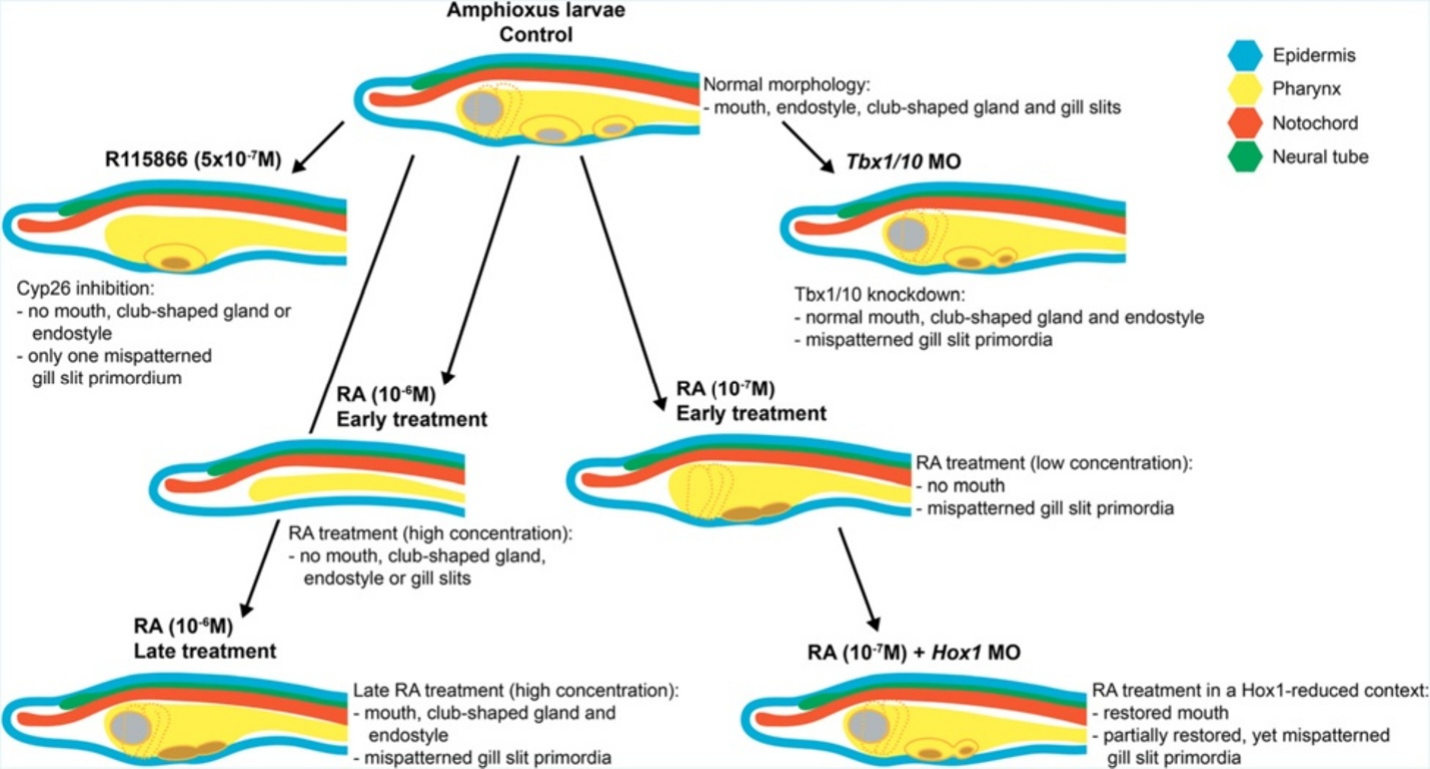
**Diagrammatic summary of the morphological defects induced by pharmacological treatments and gene knockdown in the developing amphioxus pharynx.** The effects are described in the pharynx of larvae at 36 hours post fertilization (hpf), which are characterized by a well-developed mouth (gray circle), an endostyle (anterior dotted lines), a club-shaped gland (posterior dotted lines) and two gill slit primordia (yellow ovals) with two gill slits (gray oval). Indicated are the results obtained from treatments with the Cyp26 inhibitor R115866, with retinoic acid (RA) at different developmental stages and at different concentrations, from injections of a *Tbx1/10*-specific antisense morpholino oligonucleotide (MO) and from the combination of RA treatments with the injection of a *Hox1*-specific MO. Mispatterned gill slit primordia with malformed gill slits are indicated as oval structures with brown centers. The results obtained with the retinoic acid receptor (RAR) antagonist BMS009 are not shown.

Our results indicate that, in amphioxus, RA signaling must be suppressed in the anterior portion of the gastrula for proper specification of the pharynx and that it must also be finely regulated during the neurula stage for proper partitioning of the pharynx into pharyngeal arches and pouches (Figure 7). RA signaling is controlled at several levels. The first is synthesis. Amphioxus has seven duplicates of *Aldh1/2* genes, at least some of which probably encode RA synthesizing enzymes. At the neurula stage, they are all expressed in the posterior half of the embryo [38]. However, amphioxus embryos are quite small, and RA readily diffuses through cell membranes. Therefore, RA is most likely able to diffuse anteriorly in the embryo. The second level of RA regulation is degradation by the three Cyp26 enzymes. While *Cyp26-2* is conspicuously expressed in extreme anterior and posterior tissues, *Cyp26-1* and *Cyp26-3* expression is chiefly limited to the anterior somites [18]. We found that inhibition of Cyp26 function leads to a loss of pharyngeal structures, most severely affecting the anteriormost pharynx. Finally, the third level of RA regulation is local inhibition. Our data suggest that Tbx1/10 and RA mutually inhibit each other. Thus, *Tbx1/10* turns on in mesoderm migrating into the pharyngeal arches and in adjacent pharyngeal endoderm, indicating that RA signaling must be locally suppressed for segmentation of the pharynx into pouches and arches. Later, *TR2/4*, a competitive inhibitor of RA signaling, turns on in gill slit primordia, indicating that further suppression of RA signaling in the gill slit primordia is required for gill slit penetration.

**Figure 8:**
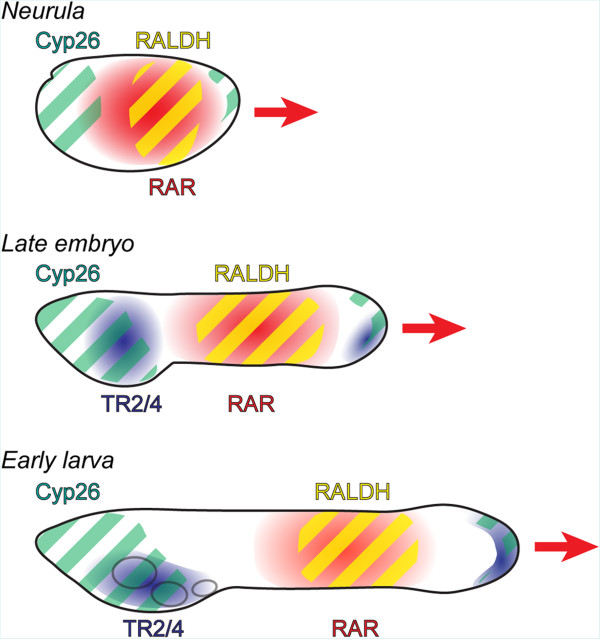
**Stage-dependent functions of retinoic acid (RA) signaling during amphioxus pharyngeal development.** Expression of the main mediators of RA signaling in the developing endoderm is indicated at three developmental stages: the neurula, the late embryo and the early larva. Shown are the domains of retinoic acid receptor (RAR), RA synthesizing enzymes (RALDH), RA degradation enzymes (Cyp26) and TR2/4, a competitive inhibitor of RA signaling. Posterior elongation of the embryo and larva in the course of development is indicated with a red arrow. As the embryo and larva elongate, the source of endogenous RA as well as the expression domain of RAR move posteriorly, hence creating a permissive environment for the patterning and development of additional pharyngeal structures, chiefly gill slits.

This work further allows us to propose a model describing the activity of RA signaling during amphioxus pharyngeal development (Figure 8). Following this model, RA signaling activity is high in the posterior half of the developing embryo, while the anterior portion is characterized by Cyp26 expression and hence by very low levels of RA. The endoderm is thus divided into anterior and posterior domains, with Cyp26 protecting the anterior domain from RA activity and with RA signaling patterning the posterior domain, including its regionalization, by defining the posterior limit of the pharynx. Expansion of the pharyngeal territory as development proceeds is accomplished through the elongation of the embryo, which concomitantly increases the distance between the posterior tip of the pharynx and the tissues producing RA and expressing RAR. This movement of RA signaling activity in the embryo hence creates a permissive, low RA environment at the posterior extremity of the pharynx allowing the patterning and formation of additional pharyngeal structures, such as gill slits. Importantly, a similar model for the activity of RA signaling has been proposed for the regulation of head mesoderm patterning in the chick embryo [39], strongly suggesting that regionalization and segmentation of the pharynx of the last common ancestor of all chordates was already dependent on RA signaling, possibly by employing an amphioxus-like molecular patterning mechanism.

It would be useful to know if our model for the first three gill slits is also applicable to the ones which subsequently form. However, it would be difficult at best to perform comparable experiments to test this. The first three gill slits of amphioxus form during the first two days of development. By 30 hpf to 36 hpf, the young larvae begin feeding [31]. However, even if not fed, they will develop normally until at least 48 hpf. Therefore, even if MO-induced gene knockdowns or manipulation of signaling pathways cause abnormal development of the gill slits and/or mouth and prevent the larvae from feeding, they will keep developing for the duration of our experiments. Once the first three gill slits develop, there is typically a lag of 2 days before the larvae begin adding more gill slits posterior to the first three. During this lag, the animals grow considerably. Starting about 4 days hpf, gill slits are added sequentially for about 2 to 3 weeks until there is a total of nine to eleven gill slits. At that point metamorphosis ensues. During metamorphosis a second row of gill slits appears on the right side dorsal to the first row, and the first row of gill slits migrates to the left side at the same time as the mouth migrates anteriorly [28, 29].

When we added RA after formation of the first three gill slits, additional gill slits did not form, but as the existing gill slits collapsed and the anus seemed to close, the animals stopped feeding (data not shown). The very first thing that happens, whenever amphioxus larvae are starved or poisoned even slightly is that all the gill slits collapse and the larvae cease feeding and cease growing. Consequently, we could not determine whether the failure of additional gill slits to form in the RA-treated larvae was due to direct effects of RA or due to starvation. These considerations severely limit the types of experiments that can be done in amphioxus to investigate whether the molecular mechanisms of gill slit formation are conserved between the first three gill slits and the ones that form later.

### Pharyngeal patterning in deuterostomes is controlled by conserved genetic mechanisms

**Figure 9:**
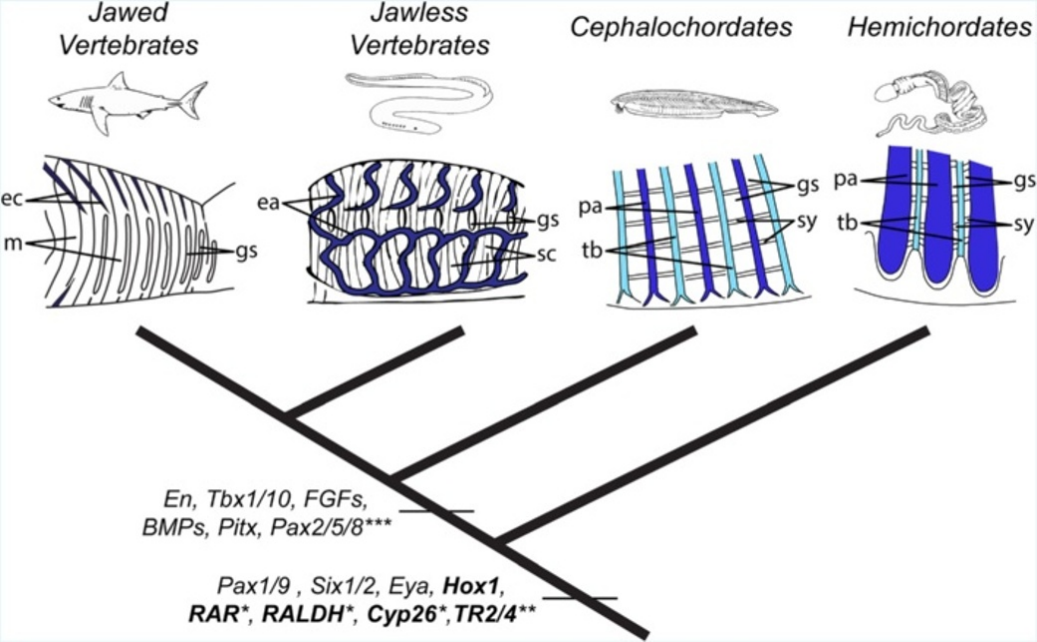
**Evolution of pharyngeal patterning in deuterostomes.** The last common ancestor of all deuterostomes possessed a segmented pharynx with alternating pharyngeal arches and tongue bars and with an extracellular collagenous skeleton secreted by endodermal cells. This ancestral pharynx was patterned by *Pax1/9*, *Six1/2* and *Eya* genes, with *Hox1* likely establishing the posterior boundary of the pharynx. The involvement of RA signaling in pharyngeal patterning in hemichordates is possible, but further work is required to validate this hypothesis. In the ancestral chordate, the pharyngeal apparatus was elaborated by *Tbx1/10*-expressing mesoderm originating from the anterior paraxial mesoderm. *Engrailed* (*En*) was co-expressed with *Tbx1/10* in the anterior somites and the gene network controlling chordate pharyngeal patterning further included *Pitx* and *Pax2/5/8* as well as fibroblast growth factor (FGF) and bone morphogenetic protein (BMP) signaling. In the vertebrate lineage, neural crest cells contribute extensively to the developing pharynx and segmentation of the head mesoderm was likely lost. Genes shown in bold are directly involved in mediating RA signaling in the pharynx. *Pharyngeal expression unknown in hemichordates; **Pharyngeal expression has not been assessed in hemichordates and vertebrates; ***Pharyngeal expression lost in terrestrial mammals. Abbreviations: ea, external branchial arches; ec, extrabranchial cartilage; gs, gill slits; mu, muscles; pa, pharyngeal arches; sc, superficial branchial constrictions; sy, synapticles; tb, tongue bar. For the sake of clarity, tunicates have not been included in the diagram.

A segmental series of gill slits is present not only in chordates, but also in hemichordates among the deuterostomes and has been described in some fossil echinoderms [40]. Comparisons of gene expression patterns indicate that at least some of the molecular mechanisms for pharyngeal specification and gill slit formation are conserved between hemichordates and chordates (Figure 9). For example, in both hemichordates and chordates, the pharyngeal endoderm expresses *Pax1/9, Six1/2* and *Eya*[13, 41–44]. In the hemichordate *Saccoglossus kowalevskii*, as in amphioxus, *Hox1* is expressed just posterior to the third primary gill slit [43], suggesting the possibility that RA signaling might act via Hox1 to establish the posterior limit of the gill slits. Hemichordate genomes encode the retinoid receptors RAR and RXR as well as homologs of vertebrate RA synthesizing (RALDH) and degrading (Cyp26) enzymes [45], but their expression and functions are still unknown. The main difference between pharyngeal patterning in hemichordates and chordates is that *Tbx1/10* is not expressed in the developing gill bars of hemichordates [43].

Comparisons between amphioxus, ascidian tunicates and vertebrates indicate that amphioxus has retained the fundamental mechanism for specification of the chordate pharynx and for partitioning it into gill slits and pharyngeal arches. For example, in both amphioxus and vertebrates, high levels of RA signaling in the middle third of the embryo establish the posterior limit of the pharynx [14–16]. Interestingly, the pharynx of juvenile ascidian tunicates might be patterned by a similar mechanism, as excess RA applied during postlarval development leads to a graded loss of the juvenile pharynx by respecification of anterior endoderm to more posterior fates [46]. Furthermore, in amphioxus, *Hox1*, which is expressed in the pharyngeal endoderm just posterior to the gills, is directly regulated by RA signaling and its knockdown causes the posterior limit of the pharynx to expand posteriorly [16]. Although this regulation is likely not conserved with ascidian tunicates [45, 47], *Hox1* expression has been described in the esophagus and posterior intestine of ascidian tunicate juveniles [47]. Taken together, the genetic mechanisms for pharyngeal specification and patterning were probably already present in the last common ancestor of all chordates and have been secondarily modified in the lineage leading to extant tunicates.

### Low RA signaling levels are required for pharyngeal segmentation and gill slit formation

In amphioxus, we found that, while adding RA at progressively later stages during the neurula causes progressively less truncation of the pharynx, the gill slits are still abnormal when RA is added at the very late neurula (24 hpf) (Figure 7). Excess RA at the mid neurula stage reduced the number of gill slit primordia, as indicated by loss of the *Six1/2* domains, while, when added at the very late neurula, RA results in strong reduction or loss of *Tbx1/10* and *Pax2/5/8* expression in the gill bars and gill slit primordia, respectively. Furthermore, when Hox1 activity is reduced, exogenous RA specifically disrupts gene expression associated with the gill slit primordia. Altogether, these data demonstrate that RA signaling must be kept low for gill slits to form in amphioxus and suggest that this function of RA is independent of Hox1. Similarly, while adding RA to lamprey embryos at the gastrula stage causes truncation of the pharynx, delaying addition until the neurula stage restores the third through seventh pharyngeal pouches, but nonetheless results in an anterior-posterior compression of the pharynx, which is accompanied by occasional fusion of adjacent arches as well as a likely absence of the endostyle [23, 24].

RA signaling interacts with Tbx1/10 and Pitx in both the amphioxus and vertebrate pharynx. In amphioxus, exogenous RA applied during the neurula stage disrupts pharyngeal segmentation and reduces *Tbx1/10* expression in the pharyngeal arches and *Pitx* expression around the mouth. Not surprisingly, inhibition of Cyp26 enzymes also downregulates *Tbx1/10* and causes the loss of a gill slit primordium. Similarly, in the lamprey, RA added during the neurula stage reduces the overall number of pharyngeal arches and induces fusion of adjacent arches [23, 24]. Expression of lamprey *Tbx1/10* and *Pitx* genes was not determined in these embryos. The effects of RA on pharyngeal segmentation have not yet been assessed in shark embryos. For other gnathostomes, *Cyp26c1* is expressed in the anterior head mesoderm of chicken [39] and its inhibition downregulates *Tbx1*. Furthermore, the caudal pharyngeal arches are lost and the pharyngeal endoderm does not segment correctly [48]. Addition of RA has a similar effect, inhibiting *Tbx1* expression, indicating that low RA signaling levels are required for *Tbx1* expression.

### Regulation of pharyngeal segmentation is conserved in amphioxus and vertebrates

The present study shows that RA signaling together with Tbx1/10 also has a role in partitioning of the amphioxus pharynx into pouches and arches (Figure 7). This is highly conserved with vertebrates. In the lamprey, as in amphioxus, *Tbx1/10* is expressed in the mesodermal core and endoderm in each of the pharyngeal arches, including the mandibular arch, as well as in the upper and lower lips, which develop from the mandibular arch [49]. Similarly, in the shark, *Tbx1* is expressed in a striped pattern in the pharyngeal mesoderm and endoderm, in the wall of the hyoid head cavity and in head mesoderm [7]. Interestingly, even though in the shark, as in all gnathostomes, the somites, which give rise to paraxial muscles, extend anteriorly only as far as the posterior hindbrain, the more anterior head mesoderm of the embryo is clearly segmented and extends sheets of mesoderm ventrally into the pharynx [9, 50]. This is reminiscent of the *Tbx1/10*-expressing amphioxus somites extending to the anterior tip of the embryo and giving rise to sheets of *Tbx1/10*-expressing mesoderm that grow into the pharynx [9]. Some of the *Tbx1*-expressing head mesoderm in gnathostomes develops into the extrinsic eye muscles, which is in agreement with the theory that segmentation of the head mesoderm, as well as of the pharynx, is conserved in amphioxus and vertebrates, with the anterior somites of the ancestral chordate evolving into head muscles in vertebrates and not differentiating as paraxial muscle as they do in amphioxus.

*Pitx* genes are expressed anteriorly to *Tbx1/10* in amphioxus as well as in vertebrates. In amphioxus, *Pitx* is expressed on the left side of the body in somites as well as in mesoderm and endoderm, extending ventrally into the pharynx and becoming localized to endoderm and ectoderm around the mouth, which has been proposed to be a modified gill slit [29]. Similarly, in the shark, *Pitx2* is expressed in the ectoderm near the mouth as well as in the walls of the hyoid and mandibular head cavities [7], while in the lamprey *PitxA* is expressed in the pre-mandibular mesoderm (head cavity), in ectoderm around the mouth, pharyngeal endoderm and in the ventral portion of the anteriormost somite [51]. In the chick, *Pitx2* is expressed in the ventral ectoderm of the head and in the first pharyngeal pouch as well as in precursors of the extra-ocular and mandibular arch muscles [39].

*Tbx1/10* and *Pitx* genes appear to synergize in patterning the head in both amphioxus and vertebrates. Our results show that, in amphioxus, knockdown of *Tbx1/10* does not affect the expression pattern of *Pitx*, but may upregulate its expression. In mouse null mutants for *Tbx1, Pitx2* is downregulated [52]. In addition, mouse Tbx1 can physically interact with the C-terminus of Pitx2 and repress the ability of Pitx2 to activate promoters of several genes, including *Pitx2c*[53]. Conversely, knockdown of *Pitx2* in the zebrafish results in smaller and abnormal cartilages of the mandibular and hyoid arches and misshapen eyes [54], and deletion of *Pitx1* in mice results in downregulation of *Tbx1*[55]. In gnathostomes, Pitx2 is further known to specify head muscles and muscles derived from the first branchial arch and to regulate *Tbx1* expression [56]. Although the regulation of *Tbx1/10* expression by Pitx has not been assessed in amphioxus, it is tempting to speculate that functional interactions of Tbx1/10 and Pitx are required for mediating pharyngeal patterning in both amphioxus and vertebrates.

### The vertebrate head mesoderm evolved from a segmented ancestor

The first sign of pharyngeal segmentation in amphioxus is the downregulation of *Pax1/9* in the pharyngeal endoderm and the simultaneous striped expression of *Tbx1/10* in the sheets of mesoderm that migrate in between the pharyngeal endoderm and ectoderm [9]. Thus, it is likely that the anterior somites and their ventral extensions, which constitute the head mesoderm in amphioxus, are pivotal in instructing pharyngeal segmentation. In gnathostomes, it has been proposed that pharyngeal segmentation is driven by expression of genes, such as *Nkx2.5*, *Wnts*, *FGFs* and *BMPs*, expressed in the head mesoderm prior to segmentation of the pharyngeal pouches [39, 57]. These genes are also expressed in the amphioxus somites, and it will be interesting to see, if in addition to the permissive role of low RA signaling in the pharynx, segmental expression of these genes in the somites is also required for pharyngeal segmentation in amphioxus. If so, it would lend additional support to the hypothesis that the anterior somites of an ancestral chordate gave rise to the head mesoderm of vertebrates and that the highly disputed somitomeres seen in gnathostome embryos may be the evolutionary equivalent of these amphioxus somites [4]. This idea has been highly contested. One school of thought is that, although the head cavities in the lamprey evolved from the anterior somites of an amphioxus-like ancestor, those of the shark, which are formed by schizocoely, are the result of a gnathostome innovation [7]. To explain similar patterns of gene expression in head structures considered morphologically non-homologous [58], the concept of heterotopy has been invoked, that is, the idea that homologous structures can alter their position during evolution and, therefore, that structures in comparable locations in different organisms may not be homologous [59]. Thus, expression of homologous genes in similar places may not indicate homologous tissues or organs [58]. The alternative view is that not only does conserved expression of genes, including *Pax1/9*, *Six1/2* and *Eya*, in the pharyngeal endoderm of hemichordates, amphioxus, tunicates, lampreys and gnathostomes indicate that a segmented pharynx was present in the common ancestor of hemichordates and chordates, but that the anterior, enterocoelic somites of amphioxus, the head cavities of the lamprey and shark and the jaw and eye muscles of bony gnathostomes are homologous [9].

Our present results, revealing striking similarities in the regulation of pharyngeal patterning between amphioxus and vertebrates, add to the body of evidence that heterotopy probably does not explain similar gene expression in head mesoderm of amphioxus and gnathostomes (Figure 9). The conserved expression of *Tbx1* in head mesoderm of gnathostomes and somites of amphioxus and the similar effects of its knockdown in both groups argue for the common evolutionary ancestry of gnathostome head mesoderm and amphioxus somites. Comparisons of the roles of additional genes segmentally expressed in both the anterior somites and mesoderm migrating into the pharyngeal arches in amphioxus with those expressed in both the early head mesoderm and developing pharynx of gnathostomes could lend additional support to the hypothesis that both the pharynx and the anterior somites of an amphioxus-like ancestral chordate evolved into the pharynx and head mesoderm of vertebrates.

## Conclusions

In this manuscript, we have used a combination of pharmacological treatments and morpholino-induced gene knockdown to study RA signaling functions during pharyngeal development of the cephalochordate amphioxus. The results allowed us to define distinct phases of RA activity in the amphioxus pharynx, mediating, for example, the anterior-posterior regionalization of the endoderm as well as the patterning and formation of pharyngeal structures. We were further able to show that Tbx1/10 is required for amphioxus gill slit development and that a reduction of Tbx1/10 activity in the pharyngeal arches has a similar effect as late RA treatments. These data suggest that segmentation of the amphioxus pharynx requires mutual inhibition of RA signaling and Tbx1/10 function. Given that similar molecular mechanisms control the patterning and segmentation of the vertebrate head, the genetic mechanisms involving RA and Tbx1/10 for partitioning the pharynx into pharyngeal pouches were probably already present in the ancestral chordate and hence precede the evolutionary elaboration of neural crest. Finally, comparisons of our results from amphioxus with data from other deuterostomes indicate that at least some of the molecular components controlling pharyngeal patterning are conserved in hemichordates and chordates, which strongly suggests that the genetic mechanisms for pharyngeal segmentation predate the origin of chordates.

## Electronic supplementary material

Additional file 1: Figure S1: An antisense morpholino oligonucleotide (MO) targeting the amphioxus *Tbx1/10* sequence suppresses translation of the amphioxus *Tbx1/10* gene *in vitro*. Each lane contains 200 ng of amphioxus *Tbx1/10* expression plasmid. While 500 ng and 2,000 ng of the amphioxus *Tbx1/10* MO efficiently block the translation of *Tbx1/10* mRNA, the equivalent amounts of control MO do not affect the *in vitro* translation of *Tbx1/10* mRNA. The arrows indicate the Tbx1/10 protein band. (TIFF 467 KB)

Additional file 2: Table S1: Stage-dependent effects of retinoic acid (RA) and RAR antagonist (BMS009) on the formation of pharyngeal structures in amphioxus. The proportion of larvae characterized by normal pharynx length and by the presence of mouth, endostyle, club-shaped gland, gill slit primordia and gill slits at 36 hours post fertilization (hpf) is indicated. Larvae were treated with 10^-6^ M RA or 2 × 10^-6^ M BMS009 from 16 hpf, 20 hpf or 24 hpf. (DOCX 80 KB)

## Abbreviations

CNS: central nervous system
CYP26: cytochrome p450 family 26
DIC: differential interference contrast
DMSO: dimethylsulfoxide
hpf: hours post fertilization
MO: morpholino oligonucleotide
RA: retinoic acid
RALDH: retinaldehyde dehydrogenase
RAR: retinoic acid receptor
RXR: retinoid X receptor.
