# Supplementary figures and images for: Roles of retinoic acid and Tbx1/10 in pharyngeal segmentation: amphioxus and the ancestral chordate condition
Source: EvoDevo. 2014 Oct 9;5:36. doi: 10.1186/2041-9139-5-36 (PMC4320481; doi:10.1186/2041-9139-5-36)

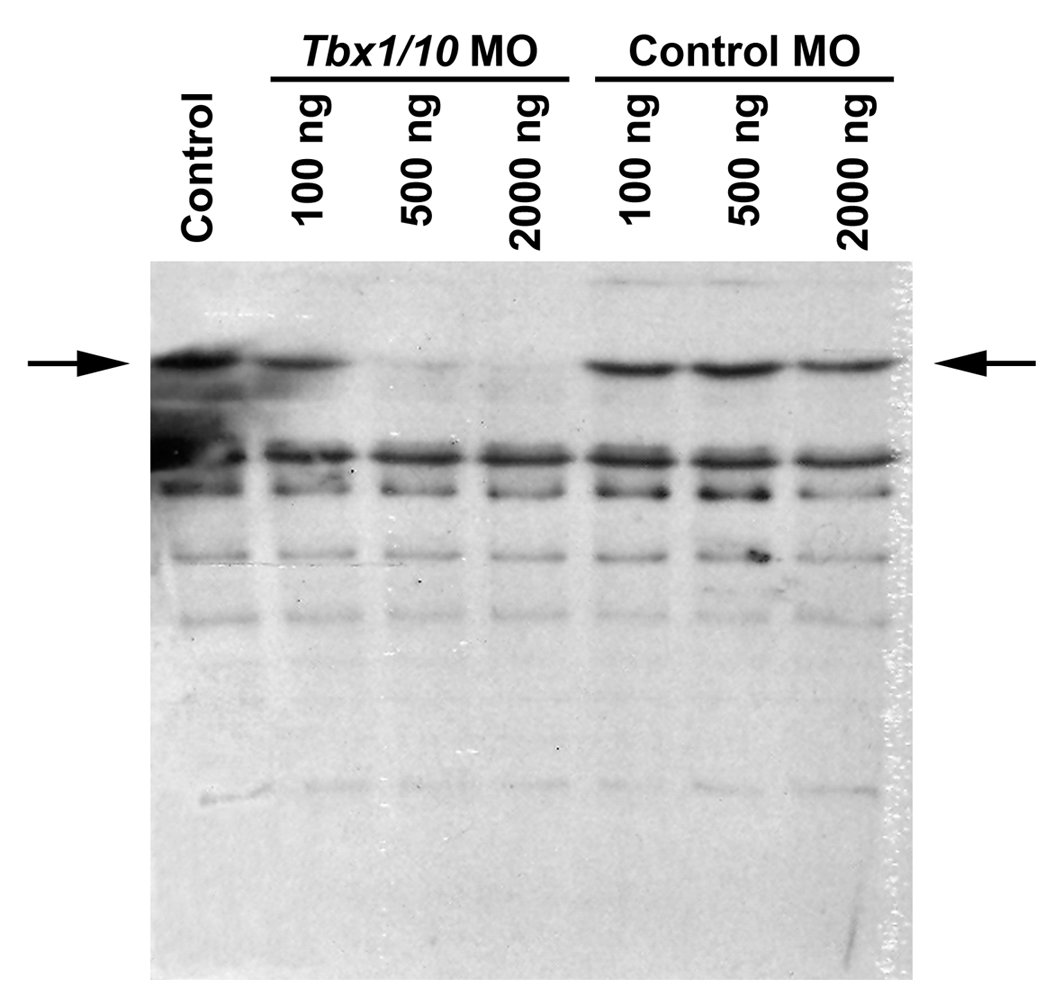

Supplement: Supplementary file 1 — Additional file 1: Figure S1: An antisense morpholino oligonucleotide (MO) targeting the amphioxus Tbx1/10 sequence suppresses translation of the amphioxus Tbx1/10 gene in vitro. Each lane contains 200 ng of amphioxus Tbx1/10 expression plasmid. While 500 ng and 2,000 ng of the amphioxus Tbx1/10 MO efficiently block the translation of Tbx1/10 mRNA, the equivalent amounts of control MO do not affect the in vitro translation of Tbx1/10 mRNA. The arrows indicate the Tbx1/10 protein band. (TIFF 467 KB) [file 13227_2014_128_MOESM1_ESM.tiff]
