# Supplementary material for: Roles of retinoic acid and Tbx1/10 in pharyngeal segmentation: amphioxus and the ancestral chordate condition
Source: EvoDevo. 2014 Oct 9;5:36. doi: 10.1186/2041-9139-5-36 (PMC4320481; doi:10.1186/2041-9139-5-36)
Supplement: Supplementary file 2 — Additional file 2: Table S1: Stage-dependent effects of retinoic acid (RA) and RAR antagonist (BMS009) on the formation of pharyngeal structures in amphioxus. The proportion of larvae characterized by normal pharynx length and by the presence of mouth, endostyle, club-shaped gland, gill slit primordia and gill slits at 36 hours post fertilization (hpf) is indicated. Larvae were treated with 10-6 M RA or 2 × 10^-6^ M BMS009 from 16 hpf, 20 hpf or 24 hpf. (DOCX 80 KB) [file 13227_2014_128_MOESM2_ESM.docx]

**Supplementary Table S1. Stage-dependent effects of retinoic acid (RA) and RAR antagonist (BMS009) on the formation of pharyngeal structures in amphioxus.** The proportion of larvae characterized by normal pharynx length and by the presence of mouth, endostyle, club-shaped gland, gill slit primordia and gill slits at 36 hours post fertilization (hpf) is indicated. Larvae were treated with 10^-6^ M RA or 2x10^-6^ M BMS009 from 16 hpf, 20 hpf or 24 hpf.

| Treatment | | Pharynx length | Mouth | Endostyle | Club-shaped gland | Gill slit primordia | Gill slits |
| --- | --- | --- | --- | --- | --- | --- | --- |
| Control | | 16/17 | 17/17 | 17/17 | 17/17 | 17/17 | 17/17 |
| RA | 16h-36h | 0/20 | 0/20 | 1/20 | 0/20 | 4/20 | 0/20 |
|  | 20h-36h | 0/19 | 0/19 | 18/19 | 10/19 | 19/19 | 0/19 |
|  | 24h-36h | 12/17 | 15/17 | 17/17 | 17/17 | 17/17 | 9/17 |
| BMS009 | 16h-36h | 9/20 | 20/20 | 17/20 | 17/20 | 19/20 | 10/20 |
|  | 20h-36h | 12/19 | 18/19 | 18/19 | 18/19 | 19/19 | 10/19 |
|  | 24h-36h | 19/20 | 19/20 | 19/20 | 19/20 | 20/20 | 16/20 |
